# A Manual of Physiology

**Published:** 1896-09

**Authors:** 


### Reviews of Books.

**A Manual of Physiology.** By G. N. Stewart, M.D. Pp. 796. London: Baillière, Tindall and Cox. 1895.

This is a carefully-written text-book of Physiology, embodying the results of the latest research in a subject which has developed so rapidly within the last few years that it is becoming more and more difficult to compress the necessary matter into a volume of moderate size. The author has, however, well fulfilled the difficult task he has undertaken, and succeeded in placing before his readers a clear outline of both theoretical and practical physiology; and has moreover included a great deal of histology. The latter is well illustrated by drawings and coloured plates; and the diagrams throughout the book are helpful and well done.

At the end of each chapter is a practical exercise which will be useful to students, and still more perhaps to demonstrators who have not had a great deal of experience in the laboratory. Some of these exercises are, almost necessarily, incomplete; and some require further explanation. But the amount of matter is so great, that too great conciseness is doubtless here and there unavoidable. Perhaps the most valuable feature is this blending of practical exercises with the statement of physiological facts; for it is of the greatest importance that students should realise from the first that physiology is nowadays an experimental science, and that laboratory work is an actual necessity. The chapters on muscle and nerve may be cited as good examples of this judicious combination, and we notice with pleasure the pains that have been taken to render these difficult subjects clear to the reader. The special senses are brought well up to date, but we could wish that smell and taste had received a little more space. The last chapter is a short account of the chief phenomena of reproduction, which may serve as an introduction to the subject, for it is too short to be of much service.

The style is clear and straightforward; there is nothing to be specially noted about it. The type is good, and there are very few errors. We regret that the index is imperfect. For example, there is nothing under the letter T; and in such a book, which will, we hope, be extensively used for reference, there should be every facility for quickly finding any special subject. This is a fault which can easily be remedied.

